# Subtypes in Patients Taking Prescribed Opioid Analgesics and Their Characteristics: A Latent Class Analysis

**DOI:** 10.3389/fpsyt.2022.918371

**Published:** 2022-07-08

**Authors:** Christian Rauschert, Nicki-Nils Seitz, Sally Olderbak, Oliver Pogarell, Tobias Dreischulte, Ludwig Kraus

**Affiliations:** ^1^Department of Epidemiology and Diagnostics, IFT Institut Für Therapieforschung, Munich, Germany; ^2^Department of Psychology, University of Arizona, Tucson, AZ, United States; ^3^Department of Psychiatry and Psychotherapy, Ludwig-Maximilians-Universität, Munich, Germany; ^4^Department of General Practice and Family Medicine, Ludwig-Maximilians-Universität, Munich, Germany; ^5^Department of Public Health Sciences, Centre for Social Research on Alcohol and Drugs, Stockholm University, Stockholm, Sweden; ^6^Institute of Psychology, ELTE Eötvös Loránd University, Budapest, Hungary

**Keywords:** opioid analgesics, opioid use disorder, prescription, latent class analysis, epidemiological survey, DSM-5

## Abstract

**Background:**

Owing to their pharmacological properties the use of opioid analgesics carries a risk of abuse and dependence, which are associated with a wide range of personal, social, and medical problems. Data-based approaches for identifying distinct patient subtypes at risk for prescription opioid use disorder in Germany are lacking.

**Objective:**

This study aimed to identify distinct subgroups of patients using prescribed opioid analgesics at risk for prescription opioid use disorder.

**Methods:**

Latent class analysis was applied to pooled data from the 2015 and 2021 Epidemiological Survey of Substance Abuse. Participants were aged 18–64 years and self-reported the use of prescribed opioid analgesics in the last year (*n* = 503). Seven class-defining variables based on behavioral, mental, and physical health characteristics commonly associated with problematic opioid use were used to identify participant subtypes. Statistical tests were performed to examine differences between the participant subtypes on sociodemographic variables and prescription opioid use disorder.

**Results:**

Three classes were extracted, which were labeled as *poor mental health group* (43.0%, *n* = 203), *polysubstance group* (10.4%, *n* = 50), and *relatively healthy group* (46.6%, *n* = 250). Individuals within the poor mental health group (23.2%, *n* = 43) and the polysubstance group (31.1%, *n* = 13) showed a higher prevalence of prescription opioid use disorder compared to those of the relatively healthy group.

**Conclusion:**

The results add further evidence to the knowledge that patients using prescribed opioid analgesics are not a homogeneous group of individuals whose needs lie in pain management alone. Rather, it becomes clear that these patients differ in their individual risk of a prescription opioid use disorder, and therefore identification of specific risks plays an important role in early prevention.

## Introduction

The use of opioid analgesics (OA) is an established component of adequate pain therapy in palliative and acute medicine for patients with severe pain (1). Over the last 25 years, an increase in OA prescriptions can be observed not only internationally in the most affected countries such as the US (2) or Canada (3), but also in Germany (4–7). Based on analyses of German health insurance data, long-term prescriptions (duration ≥3 months) increased by about four percentage points between 2006 and 2015 (8). Studies showed that most of these prescriptions are for patients with chronic non-cancer pain (4), despite evidence on the effectiveness in this patient group is lacking (9). Owing to their pharmacological properties, the use of OA carries the risk of abuse and dependence (10, 11). In Germany, the 1-year prevalence of prescription opioid use disorder (pOUD) among individuals reporting use of opioids prescribed to them by their providers is currently estimated at 21.2% (12). Even though Germany may not currently be affected by an opioid epidemic (6, 13), the high prevalence of pOUD is concerning. Abuse and dependence are associated with a wide range of personal, social, and medical problems and therefore should be prevented (14). The German S3 guideline “Long-term administration of opioids for non-tumor pain” takes this into account and provides evidence-based recommendations to clinicians for appropriate treatment (15).

Among the behavioral, mental, and physical health conditions that are associated with pOUD are co-occurring alcohol use disorder, polysubstance use of illicit drugs (16, 17), depression as well as other psychiatric disorders (12, 16, 18, 19). Although many studies have identified associations between pOUD and single risk factors, little is known about the existence of specific patient subgroups and their individual risk of pOUD. Data-driven approaches to identify such groups have mainly been applied in the US (20–22) but are lacking in Germany. Identifying distinct subtypes of patients taking prescribed OA and their individual risk of pOUD may inform decisions around the need for and length of opioid use as well as strategies to prevent pOUD in patients with severe non-cancer pain.

The aim of this study was to (1) identify distinct subgroups of individuals who used prescribed OA in the past year based on behavioral, mental, and health characteristics and (2) compare the subgroups by risk of pOUD.

## Methods

### Study Design and Sample

The data used in this study are from the Epidemiological Survey of Substance Abuse (ESA), a representative cross-sectional study of Germany's general population on the use of substances that is conducted every 3 years [for a detailed description of the methodology and design of the single studies, see (23)]. The ESA sample was drawn from the 18- to 64-year-old German-speaking population living in private households. Random sampling was carried out using a two-stage selection procedure. First, 254 “sample points” (i.e., cities, districts, municipalities) were selected proportionally to their population size. Then individuals living in these communities were randomly selected based on personal addresses from population registers. Data were collected through the use of a questionnaire (paper-pencil, telephone interviews, or online). A weight correction (age, gender, education, federal states, and district size class) based on the Iterative Proportional Fitting Algorithm was used to weight the data (24).

We pooled data from two waves of the ESA—years 2015 (*n* = 9,204) and 2021 (*n* = 9,046)—in which information about OA was available, resulting in a total of 18,250 cases (see section Data Analysis for justification of pooling). Of the total sample 10,199 women (55.9%), 8,038 men (44.0%), and 13 who selected other (0.07%) participated in this study with an average age of 38.4 years (*SD* = 0.12). The ESA was approved by the ethics committee of the German Psychological Society (DGPs; Reg.-No: GBLK06102008DGPS).

For the present analysis, only individuals reporting the use of OA within the last 12 months and receiving their medication via a prescription from a medical doctor were included. For the remainder of the manuscript, we refer to these persons as patients.

### Variables for Latent Class Analysis (pOUD Risk Factors)

A review of the literature on risk factors identified several behavioral, mental, and health characteristics highly associated with pOUD, which were collected in our survey: hazardous alcohol consumption (25), daily smoking (26), consumption of cannabis (27), consumption of other illicit drugs (amphetamines, ecstasy, LSD, heroin and other opioids, cocaine, crack, hallucinogenic mushrooms) (28, 29), depression, psychological treatment (16, 17, 19, 30), and poor physical health status (31). All variables were dichotomized (yes/no). Hazardous alcohol consumption was assessed using the German version of the Alcohol Use Disorder Identification Test, a screening instrument for alcohol-related disorders with a cut-off value of 8 points out of a possible total of 40 points for having a positive screen (32–34). Daily smoking was defined as the consumption of at least one cigarette per day in the last 30 days. Self-reported information about the consumption of cannabis as well as of other illicit drugs was assessed for the last 12 months. Screening for mental health conditions such as depression as well as information on whether a participant was in treatment for mental health problems or had been diagnosed with a mental disorder was achieved using questions from the Munich Composite International Diagnostic Interview (35, 36). According to the World Health Survey (37), self-rated physical health status was measured using a five-point Likert scale ranging from 1, very good, to 5, very bad. To dichotomize the variable poor health status, the first three points of the scale were summarized and coded as “no,” whereas the last two points of the scale were summarized and coded as “yes.” These variables were used to identify distinct subgroups of individuals reporting the use of prescribed OA.

### Selection of External Factors Related to Extracted Classes

The variable of highest interest, pOUD, was assessed using the written version of the Munich Composite International Diagnostic Interview (35, 36). According to the fifth version of the Diagnostic and Statistical Manual of Mental Disorders (DSM-5) (38), an opioid use disorder is defined as having occurred if at least two out of 11 criteria have been met in the last 12 months. However, in patients taking prescribed OA under appropriate medical supervision, only nine criteria are applicable as recommended by the DSM-5. Therefore, the criteria “tolerance” and “withdrawal” symptoms are excluded from the DSM-5 diagnosis and were not considered in the analyses in this paper. Further variables to test for class membership were sociodemographic variables including age (metric), gender (male/female; as none of the participants who selected “other” reported OA use), education (low/middle/high), currently employed (yes/no), and net household income (OECD modified equivalence scale) below the poverty threshold (yes/no). The variable education was categorized into three groups according to the International Standard Classification of Education (ISCED) as follows: low (ISCED 1 and ISCED 2), middle (ISCED 3 and ISCED 4), and high (ISCED 5) (39).

### Data Analysis

To reach a sample size of *n* ≥ 500, which is sufficiently powered for latent class analysis (LCA) (40–42), the data from two ESA waves, years 2015 and 2021, were pooled. Before the data was pooled, it was tested whether the mean levels (or categorical proportions) and relations between all variables included in the LCA, χ^2^ tests, and *t*-tests were comparable between the waves. We found no statistical difference in the correlations between all variables between the waves. Differences in means (continuous variables) and proportions (categorical variables) were tested using independent *t*-tests and χ^2^ tests, respectively (for detailed information see Supplementary Table 1). No statistically significant differences were found (all *p*-values ≥ 0.132) except for the variable mode of administration (i.e., paper-pencil, telephone, or internet-based) (*p*-value < 0.01). However, this difference was expected due to an increased internet use from 2015 to 2021: 38.4% of the participants answered the ESA questionnaire online in 2015 whilst in 2021 61.6%.

Seven class-defining variables, as described above, were used to delineate subgroups of patients taking prescription OA using LCA. The LCA is a probabilistic model-based approach that uses multivariate categorical data as inputs to identify unobserved, mutually exclusive classes (subgroups of individuals) in a certain population. Membership of an individual in a specific class is based on similarities in individual response behavior to some chosen set of observed variables by calculating class membership probabilities (43, 44). To determine the number of latent classes, five alternative models were run, ranging from one to five classes. A complex mixture term was needed to avoid biased results resulting from the two-stage selection procedure of the ESA. To choose the best model with an optimum number of classes, goodness-of-fit-criteria such as the Akaike Information Criterion (AIC), the Bayesian Information Criterion (BIC), the adjusted Bayesian Information Criterion (aBIC) as well as entropy were used to compare the five models. High entropy, as well as low values of AIC, aBIC, and BIC, indicate a good class solution, where the BIC was considered the best indicator (40, 45). To evaluate the discriminatory power of the models, average class assignment probabilities were determined (45). The selection of the final model was also based on the researchers' assessment of the interpretability of the results. The LCA estimation was conducted in MPlus 6.12 (46) using maximum likelihood estimation with robust standard errors.

To identify associations between class membership and pOUD as well as sociodemographic factors (described above), statistical tests were performed. Testing for differences between the identified groups was done with χ^2^ tests for categorical variables and *t*-tests for continuous variables. An alpha level of 0.05 was considered for statistical significance. All analysis was performed using Stata 15.1 SE (Stata Corp LP; College Station, TX, USA) (47).

## Results

### Analysis Sample

Data were available for 537 people who reported using prescribed OA in the past year, of which 34 individuals were excluded because of missing values on key variables: pOUD (21 individuals), unemployed (10 individuals), and education according to the ISCED (3 individuals). The final analysis sample was comprised of 503 individuals.

### Prevalence Distribution of Class-Defining Variables

Information about the weighted prevalence proportions (with listwise deletion of missing data) of the seven class-defining variables is shown in Table 1. Depression (60.2%), as well as daily smoking (32.2%), were the most prevalent indicators, whereas the use of other illicit drugs (9.2%) and the use of cannabis (12.9%) showed the lowest prevalence proportions. About one-fifth (18.3%) of the respondents met the criteria for hazardous alcohol consumption, and one quarter (26.7%) reported being treated for mental health problems or a diagnosed mental disorder.

**Table 1:** Weighted prevalence proportions of class-defining variables (*n* = 503).

**Indicators**	***n***	**%**	**95% CI**
Hazardous alcohol use	107	18.3%	[14.5; 22.9]
Daily smoking	128	32.2%	[26.9; 38.0]
Cannabis use	65	12.9%	[9.5; 17.4]
Other illicit drug use	37	9.2%	[6.2; 13.5]
Depression	290	60.2%	[54.8; 65.4]
Psychological treatment	138	26.7%	[22.2; 31.7]
Poor health	123	29.9%	[25.2; 35.0]

*% = weighted prevalence for age, region, gender and education; 95% CI, 95% confidence interval; Note: Listwise deletion of missing data*.

### Identifying Subgroups With LCA

A review of the goodness-of-fit indicators revealed contradictory recommendations regarding the appropriate number of classes. We ultimately decided on the three-class solution because it had the best fit according to two of the fit indices (BIC and aBIC), a better distribution of class prevalence (average class assignment probability was >0.8), and the subtypes were associated with meaningful and clearly distinct subgroups values (Table 2). The assumption of conditional independence in all three classes was met (45).

**Table 2:** Goodness-of-fit-measures and class-specific response probabilities of the five investigated models for deciding the number of classes (*n* = 503).

**Number of classes**	**Goodness-of-fit measures**				**Estimated class-specific response probability**				
	**Entropy**	**AIC**	**BIC**	**aBIC**	**Class 1**	**Class 2**	**Class 3**	**Class 4**	**Class 5**
1	–	–	3701.803	–	1.000	–	–	–	–
2	0.900	3531.960	3595.269	3547.657	0.910	0.985	–	–	–
3	0.723	3409.778	3506.851	3433.847	0.934	0.865	0.897	–	–
4	0.735	3407.447	3538.285	3439.889	0.834	0.842	0.906	0.825	–
5	0.702	3404.796	3569.399	3445.610	0.879	0.686	0.780	0.937	0.875

*AIC, Akaike Information Criterion; BIC, Bayesian Information Criterion; aBIC, adjusted Bayesian Information Criterion*.

**Figure 1:**
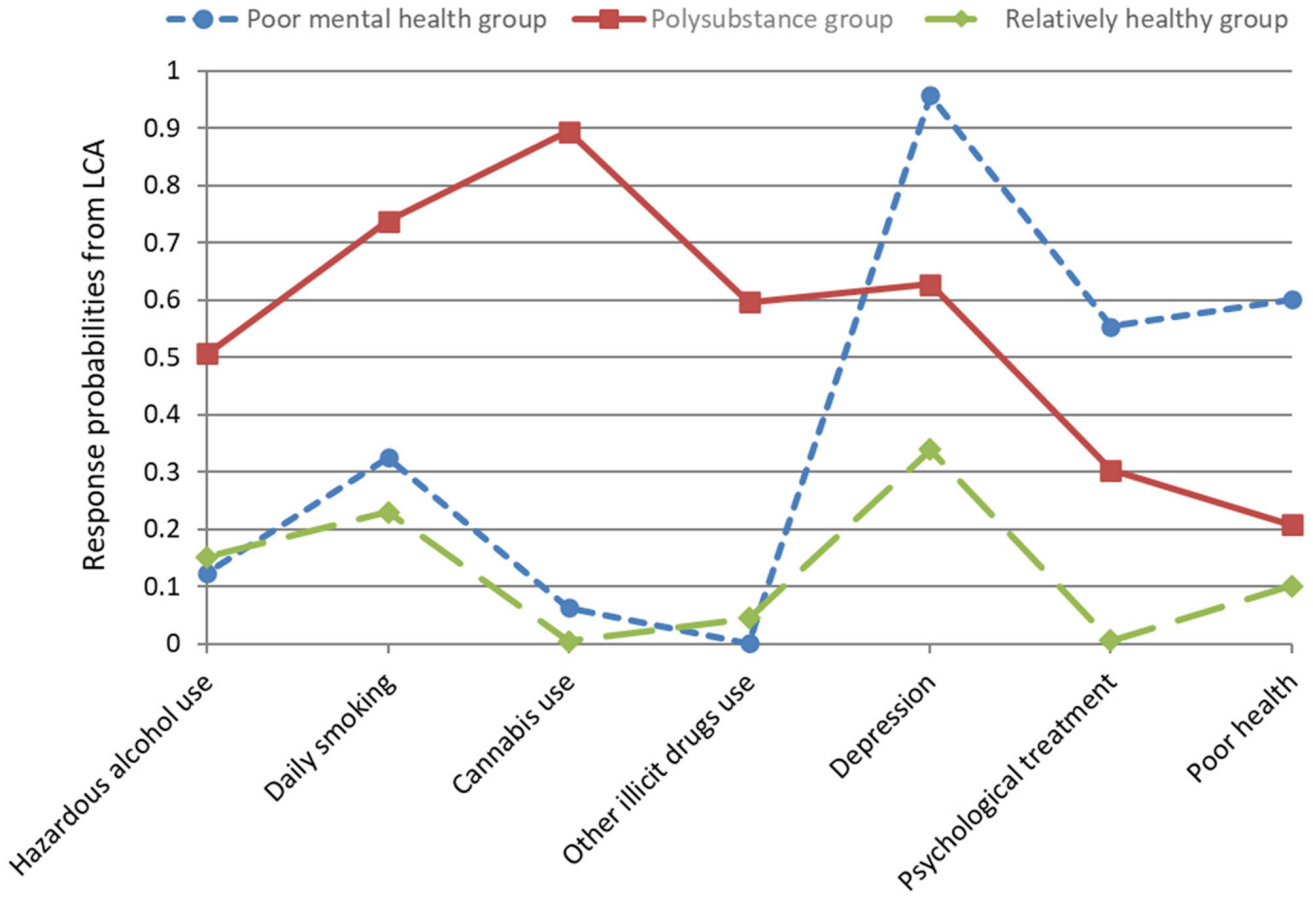
Estimated class-specific response probabilities for seven pOUD risk factors. A high score indicates a high probability of a particular risk factor.

Estimated class-specific response probabilities indicating patterns of three distinct subgroups of individuals using prescription OA are presented in Figure 1. The first class (43.0%, *n* = 203) showed the highest value for poor health (60.1%) of all classes and very low values for cannabis use (6.3%) and other illicit drug use (0.01%). On account of the highest values for depression (95.7%) and psychological treatment (55.4%) compared with the other classes and low to moderate values in all other class-defining variables, this class was characterized as the “*poor mental health group*”.

Response probabilities of individuals in class 2 (10.4%, *n* = 50) were very high in all substance use variables, with cannabis use showing the highest value (89.4%). Although patients in this class showed a high value for depression, this class was defined as the “*polysubstance group”*.

Class 3 (46.6%, *n* = 250) comprised individuals who were less likely to have poor health (10.1%), less likely to be in psychological treatment (0.5%), and less likely to be engaging in hazardous alcohol use (15.2%), cannabis use (0.4%), or the use of other illicit drugs (4.5%). They also had moderate values for daily smoking (23.0%) and depression (33.9%). Thus, the third class was labeled as the “*relatively healthy group*”.

### Baseline Characteristics of Extracted Subgroups

Information about weighted proportions of sociodemographic data and pOUD over all three subgroups is displayed in Table 3. Individuals in the *poor mental health group* were predominantly female (68.9%, *n* = 156) with an average age of 46.0 years (*SD* = 0.93), and the majority had a middle level of education (53.9%, *n* = 104). In this subgroup, 22.4% (*n* = 44) reported having a net household income below the poverty threshold and 34.4% (*n* = 71) were unemployed. Diagnostic criteria for pOUD were met in 23.2% (*n* = 43) of patients.

**Table 3:** Baseline characteristics of the extracted classes.

**Indicators**		**Total sample**	**Poor mental health group**	**Polysubstance group**	**Relatively healthy group**
% (*n*)		100.0 (503)	43.0 (203)	10.4 (50)	46.6 (250)
Age	Mean age (SD)	45.1 (0.65)	46.0 (0.93)	38.4 (1.95)	45.7 (0.85)
	*p*-value	–	0.781^a^	**0.000^a^**	–
Gender	% female (*n*)	60.0 (326)	68.9 (156)	49.0 (24)	54.2 (146)
	*p*-value	–	**0.009^a^**	0.595^a^	–
Education	% low (*n*)	18.5 (74)	18.7 (34)	47.0 (16)	11.9 (24)
	% middle (*n*)	49.1 (227)	53.9 (104)	34.4 (18)	47.9 (105)
	% high (*n*)	32.5 (202)	27.4 (65)	18.6 (16)	40.3 (121)
	*p*-value	–	0.050^a^	**0.000^a^**	–
Unemployed	% (*n*)	25.9 (133)	34.4 (71)	29.9 (11)	17.3 (51)
	*p*-value	–	**0.001^a^**	0.130^a^	–
Income below poverty threshold	% (*n*)	24.0 (93)	22.4 (44)	58.6 (17)	17.7 (32)
	*p*-value	–	0.359^a^	**0.000^a^**	–
Prescription opioid use disorder	% (*n*)	18.2 (81)	23.2 (43)	31.1 (13)	10.7 (25)
	*p*-value	–	**0.007^a^**	**0.004^a^**	–

a *Comparison of classes: reference group was the relatively healthy group. SD, standard deviation. Note: Percentages are weighted for age, gender, region and education. p-values based on χ^2^-test for categorial variables and t-test for continuous variables. Bold text denotes statistical significance with p < 0.05*.

Patients in the *polysubstance group* were on average 38.4 years old (*SD* = 1.95), and 49.0% (*n* = 24) were female. The level of education in this subgroup was predominantly low (47.0%, *n* = 16), 29.9% (*n* = 11) were unemployed, and 58.6% (*n* = 17) had an income below the poverty threshold. pOUD was detected in 31.1% (*n* = 13) of patients.

The *relatively healthy group* was marked by individuals with an average age of 45.7 years (*SD* = 0.85). More than half of all patients in this subgroup were female (54.2%, *n* = 146), and the majority had a middle level of education (47.9%, *n* = 105). The proportion of unemployed individuals in this subgroup was 17.3% (*n* = 51), and 17.7% (*n* = 32) had a net household income below the poverty threshold. pOUD occurred in 10.7% (*n* = 25) of patients.

### Differences Between Subgroups on External Factors

Individuals in the *poor mental health group* showed significantly higher proportions of pOUD compared with the *relatively healthy group* (23.2% vs. 10.7%). Moreover, patients in this subgroup were more likely to be female (68.9% vs. 54.2%) and unemployed (25.5% vs. 17.3%) compared to those in the *relatively healthy group*. A higher proportion of individuals within the *polysubstance group* had a diagnosis of pOUD compared with the *relatively healthy group* (31.1% vs. 10.7%). Additionally, members of this subgroup were more likely to be younger, with a lower level of education (47.0% vs. 11.9%) as well as a net household income below the poverty threshold (58.6% vs. 17.7%) compared with those in the *relatively healthy group*.

## Discussion

The present study investigated pooled cross-sectional data from the ESA for the years 2015 and 2021 to identify distinct subgroups of patients using prescribed OA, based on behavioral, mental, and physical health characteristics commonly considered to be relevant to problematic OA use. The study aimed to address differences in the prevalence of pOUD between the single subgroups. Latent class analysis revealed that a three-class model best fitted the data. Subgroups were labeled as *poor mental health group* (43.0%, *n* = 203), *polysubstance group* (10.4%, *n* = 50), and *relatively healthy group* (46.6%, *n* = 250). Testing for group differences indicated that individuals in the *poor mental health* subgroup as well as individuals in the *polysubstance group* showed a higher prevalence of pOUD compared to the *relatively healthy group*. Furthermore, patients in the *poor mental health group* were more likely to be female and unemployed. Against that, patients of the *polysubstance group* were more likely to be younger, with a lower level of education and a net household income below the poverty threshold. To our knowledge, this is the first study using a model-based approach to identify distinct subgroups of individuals reporting the use of prescribed OA on a population-based level in Germany. The results add further evidence to the knowledge that patients using prescribed OA are a heterogeneous group of individuals, many of whom are likely to have needs beyond pain management alone. It becomes clear that patients using prescribed OAs differ in their individual risk of pOUD.

When interpreting the findings in relation to previously published work, several of the results could be confirmed. For example, Cochran et al. also identified three subtypes of individuals using prescribed OA while analyzing community pharmacy data in southwestern Pennsylvania (20). Subgroups in this study were labeled as hazardous alcohol group, mental health group, and poor health group, and patients in the mental health group showed the highest risk of opioid misuse. Another study performing LCA among individuals using prescribed OA in the US population also identified a group with mental health issues, labeled as the depressed class (22). Patients in this class were more likely to be female which was in coincidence with the findings of the present study. In the same study, the authors found higher rates of pOUD in patients with comorbid psychopathological disorders. A link between mental disorders and a higher risk of pOUD was also found in other studies (17–19, 48, 49). These findings can be confirmed by the results of the present study, by showing a significant relationship between membership of the *poor mental health group* and a positive diagnosis of pOUD compared to the *relatively healthy group*. As in the present study individuals in the *poor mental health group* also showed signs of a higher prevalence of unemployment compared to patients in the *relatively healthy group*, this association could be confirmed by previous studies (50, 51).

As mental health problems and physical disorders often exacerbate each other and pain management, more sophisticated interdisciplinary therapy approaches are needed (20, 52, 53). Psychosocial interventions such as cognitive behavioral therapy, in combination with medical pain management, represent an important resource for patients in this subgroup (54). As individuals in this specific subgroup also showed a high prevalence of unemployment, additional linking of these patients to services in the community to address social determinants of health (e.g., vocational services) might be considered for enhancement of treatment (55). Our findings also indicated that individuals in the poor mental health group are more likely to be female, therefore gender-specific treatment programs should be considered in an integrated healthcare setting. This might lower the barriers for some individuals to access substance abuse treatment with enhancing overall treatment outcomes (56).

Identifying individuals in the *poor mental health subgroup* might enable early intervention strategies such as referral to specialized care for these patients (20, 57).

Besides mental health problems, the use of illicit drugs as well as alcohol abuse and smoking are also serious problems in pain patients taking prescription OA that are associated with a higher risk of pOUD (16, 19, 58–61). The results of the present study are in line with those of Afshar et al. who also detected a *polysubstance group* in hospitalized patients with opioid misuse (21). Patients in this subgroup also showed lower socioeconomic status, which could be confirmed by the present result of a very low net household income in the *polysubstance group*. Larger studies with sample sizes reaching from 19,000 to 26,000 individuals enrolled in substance abuse treatment programs also identified subtypes of individuals using prescribed OA with polysubstance use and therefore match our labeling for this group (62, 63). Patients in this subgroup might require access to comprehensive addiction services, such as professional addiction counseling and therapy, in addition to appropriate pain treatment. Further, the present findings indicate that patients in the *polysubstance group* differed significantly in sociodemographic factors compared with the *relatively healthy group*. Therefore, we strongly support the recommendation from the German S3 guideline “Medikamentenbezogene Störungen” [Medication-Related Disorders] for early screening for psychosocial and substance use characteristics at the very beginning of opioid therapy (64).

The present findings also identified a large group of patients using prescribed OA showing no noticeable behavioral, mental or physical health characteristics on most of the indicator variables. Due to moderate values on daily smoking and depression, this class was labeled as the *relatively healthy group*. However, these levels of depression and smoking are comparable to the general German population (65, 66). While Green et al. also identified a medically healthy subgroup in their analysis (63), individuals in the present analysis differed in low proportions of substance use (alcohol, cannabis, illicit drugs) and mental health problems. Statistical testing showed that patients in *the relatively healthy group*, relative to the other subgroups, also had the lowest proportion of a positive pOUD diagnosis (10.7%). Individuals in this subgroup reported the use of OA but hardly showed signs of poor health. It may be possible that these individuals had already overcome successful opioid therapy before answering the survey questionnaire. Assuming that pain reduction is the primary concern in these patients and appropriate medication adherence is ensured, this may lower the risk of pOUD.

### Limitations

The present findings are subject to several limitations. As the data in the study are based on self-reports, validity is strongly dependent on the response behavior of the individuals interviewed. Biases resulting from lack of memory and response tendencies resulting from social desirability can neither be quantified nor excluded. Regarding the representativeness of the results, it should be noted that, with the present study design, certain population groups are not or hardly reached. These are mainly individuals older than 64 years, homeless people as well as inmates or people who are accommodated in medical institutions (67). The last category primarily concerns palliative patients. In the case of chronic and acute diseases, the influence of possible hospitalization is quite small as the survey periods lasted about 6 months.

Future studies should provide a deeper investigation into the variables that may help to predict pOUD. For instance, the duration of exposure, information about the pain condition treated, as well as information about who was the prescriber of the OA (i.e., specialty vs. general medical prescriber). Unfortunately, these questions were not possible to address in the current paper, because this data was not collected in either ESA wave.

Because of the pooling of two cross-sectional surveys, the participation of individuals in both ESA surveys cannot be fully excluded. The two-stage random sampling design, however, essentially eliminates this possibility.

Finally, there is also the possibility that people misusing their prescribed OA were included in our analysis sample, which might affect our findings regarding patients being treated for pain conditions. Although not completely impossible, the inclusion of individuals misusing opioids under a prescription regime is very unlikely given the rather strict German prescription regulations (64).

## Conclusion

The findings of the present study add new evidence to the classification of individuals using prescribed OA and the prevalence of pOUD on a nationally representative level in Germany. Two specific subgroups with a high prevalence of pOUD, labeled as the *poor mental health group* and *polysubstance group* were identified. While individuals in the *poor mental health group* were more likely to be female and unemployed, patients in the *polysubstance group* were marked by younger age with a low level of education and a net household income below the poverty threshold. Clinicians treating patients in opioid therapy should be aware of these specific subtypes and their highly related risk of pOUD during medical history. Using data-based approaches to delineate patient subgroups from different data sources is highly recommended in further research to gain urgently needed insight into subtype characteristics. Stratification of these subtypes enables targeted early intervention from a clinical as well as a public health perspective.

## Data Availability Statement

Publicly available datasets were analyzed in this study. This data can be found here: https://www.esa-survey.de/ergebnisse/datenzugang.html.

## Ethics Statement

The studies involving human participants were reviewed and approved by German Psychological Society (DGPs). Written informed consent for participation was not required for this study in accordance with the national legislation and the institutional requirements.

## Author Contributions

CR analyzed and interpreted the data and wrote the initial draft of the manuscript. LK designed the study. All authors gave important feedback in revising the manuscript, commented on various versions of the article, and approved the final version.

## Funding

The Epidemiological Survey of Substance Abuse (ESA) was supported by funding from the German Federal Ministry of Health (project no. ZMVI1-2520DSM203).

## Conflict of Interest

The authors declare that the research was conducted in the absence of any commercial or financial relationships that could be construed as a potential conflict of interest.

## Publisher's Note

All claims expressed in this article are solely those of the authors and do not necessarily represent those of their affiliated organizations, or those of the publisher, the editors and the reviewers. Any product that may be evaluated in this article, or claim that may be made by its manufacturer, is not guaranteed or endorsed by the publisher.
