# Supplementary material for: Subtypes in Patients Taking Prescribed Opioid Analgesics and Their Characteristics: A Latent Class Analysis
Source: Front Psychiatry. 2022 Jul 8;13:918371. doi: 10.3389/fpsyt.2022.918371 (PMC9304960; doi:10.3389/fpsyt.2022.918371)
Supplement: Supplementary file 1 [file Table_1.docx]

**Table S1.** Sociodemographic- behavioral- and health-related characteristics

of individuals using prescribed opioid analgesics between the ESA years

2015 and 2021.

| Characteristics | |  | ESA 2015  (*n* = 253) |  | ESA 2021 (*n* = 250) |  |  |
| --- | --- | --- | --- | --- | --- | --- | --- |
|  |  |  | % (*n*) |  | % (*n*) |  | *p-value* |
| Age | |  |  |  |  |  | 0.310 |
|  | Mean (*SD*) |  | 44.4 (0.85) |  | 45.8 (0.96) |  |  |
| Gender | |  |  |  |  |  | 0.198 |
|  | Male |  | 36.5 (83) |  | 43.8 (94) |  |  |
|  | Female |  | 63.5 (170) |  | 56.2 (156) |  |  |
| Education | |  |  |  |  |  | 0.289 |
|  | Low |  | 20.8 (37) |  | 15.9 (37) |  |  |
|  | Middle |  | 50.2 (124) |  | 47.8 (103) |  |  |
|  | High |  | 29.0 (92) |  | 36.3 (110) |  |  |
| Unemployed | |  |  |  |  |  | 0.638 |
|  | Yes |  | 27.1 (68) |  | 24.7 (65) |  |  |
|  | No |  | 72.9 (185) |  | 75.3 (185) |  |  |
| Income below poverty threshold | |  |  |  |  |  | 0.596 |
|  | Yes |  | 25.2 (49) |  | 22.5 (44) |  |  |
|  | No |  | 74.8 (204) |  | 77.5 (206) |  |  |
| Hazardous alcohol use | |  |  |  |  |  | 0.621 |
|  | Yes |  | 17.3 (52) |  | 19.4 (55) |  |  |
|  | No |  | 82.7 (200) |  | 80.6 (194) |  |  |
| Daily smoking | |  |  |  |  |  |  |
|  | Yes |  | 36.2 (64) |  | 27.7 (64) |  | 0.132 |
|  | No |  | 63.8 (186) |  | 72.3 (184) |  |  |
| Cannabis use | |  |  |  |  |  | 0.322 |
|  | Yes |  | 11.0 (25) |  | 15.1 (40) |  |  |
|  | No |  | 89.0 (228) |  | 84.9 (207) |  |  |
| Other illicit drug use | |  |  |  |  |  | 0.790 |
|  | Yes |  | 8.8 (17) |  | 9.7 (20) |  |  |
|  | No |  | 91.2 (236) |  | 90.3 (228) |  |  |
| Depression | |  |  |  |  |  | 0.288 |
|  | Yes |  | 57.5 (130) |  | 63.2 (160) |  |  |
|  | No |  | 42.5 (122) |  | 36.8 (88) |  |  |
| Psychological treatment | |  |  |  |  |  | 0.488 |
|  | Yes |  | 25.0 (58) |  | 28.4 (80) |  |  |
|  | No |  | 75.0 (194) |  | 72.6 (170) |  |  |
| Poor health | |  |  |  |  |  | 0.682 |
|  | Yes |  | 30.9 (62) |  | 28.8 (61) |  |  |
|  | No |  | 69.1 (190) |  | 71.2 (189) |  |  |
| Mode of administration | |  |  |  |  |  | **0.000** |
|  | Telephone (CATI) |  | 33.0 (82) |  | 11.3 (26) |  |  |
|  | Paper-pencil (PAPI) |  | 43.2 (95) |  | 47.1 (121) |  |  |
|  | Internet (CAWI) |  | 23.8 (76) |  | 41.6 (103) |  |  |

n = observations; % = weighted prevalence rates; SD = standard deviation;

p-values based on χ ²-test for categorial variables and t-test for continuous variables;

Note: Bold text denotes statistical significance with p< 0.05.
